# Glomerulocystic kidney presenting as an incidental finding in an adult male with unilateral non-functioning kidney

**DOI:** 10.12861/jrip.2014.30

**Published:** 2014-12-01

**Authors:** Muhammed Mubarak, Shaheera Shakeel, Javed Iqbal Kazi

**Affiliations:** ^1^Department of Histopathology, Sindh Institute of Urology and Transplantation (SIUT) Karachi, Pakistan

**Keywords:** Glomerulocystic kidney, Hydronephrosis, Pyuria

**Implication for health policy/practice/research/medical education:**

Glomerulocystic kidney is a lesion with protean causes and clinical manifestations. Recent clinicopathological and molecular genetic analysis have shed light on the varied etiology, symptomatology, presenting features and have allowed a better classification of the disease. An accurate categorization of the lesion is of utmost importance for the proper prognostication and appropriate management of individual patients.

## Case presentation

A 50-years-old male presented with right lumbar pain since 1 year and frequency, intermittency, pyuria, and fever since 6 days. The pain was radiating to groin along with hematuria. No past surgical history of note was elicited. Family history was unremarkable for kidney disease. He was a known case of asthma. On ultrasound abdomen, his left kidney was normal, but the right showed gross hydronephrosis. Plain X-Ray abdomen and intravenous pyelography (IVP) was performed, which revealed left normal kidney, and right non-functioning kidney with gross hydronephrosis.

The laboratory examination revealed hemoglobin (Hb), 13.0 g/dl; total leukocyte count (TLC) of 11,900/cmm; neutrophils, 72%; erythrocyte sedimentation rate (ESR), 71 mmHg/first hour; serum urea, 32 mg/dl; and serum creatinine, 0.7 mg/dl. Urine analysis showed pH, 7.0; specific gravity, 1.020; albumin, +1; nitrate, trace; pus cells, plenty/HPF; red blood cells (RBCs), 4-6/HPF; and epithelial cells, 2-4/HPF. Urine culture revealed no growth.

MAG scan showed 0% functioning right kidney, left kidney was 100% functioning.

Right nephrectomy was performed. Preoperatively, gross hydronephrosis with thin cortex was observed. On gross examination, there was gross dilatation of the pelvicalyceal system with thinned out cortex (Figure 1A). A single small stone was found impacted in the upper ureter causing obstruction. Histopathological evaluation revealed marked hydronephrosis and changes of chronic non-specific pyelonephritis. In addition, the majority of the glomeruli showed cystically dilated Bowman’s spaces to more than 2-3 times normal size with small capillary tufts (Figure 1B, C). Many glomeruli were also globally sclerosed (Figure 1D). The postoperative course was uneventful and the patient is maintaining normal renal function three years after the surgical intervention.

Figure 1 A) Gross photograph of the right kidney showing gross hydronephrosis and thinned out cortex. B) Medium-power view showing many cystic glomeruli with dilated Bowman’s spaces. One normal glomerulus is also seen in the field. There is moderate tubular atrophy and interstitial inflammation. (H&amp;E, ×200). C) High-power view showing many cystic glomeruli with small shrunken glomerular tufts. There is tubular atrophy and interstitial inflammation of at least moderate degree. (H&amp;E, ×400). D) High-power view showing cystic glomeruli with small shrunken glomerular tufts. One glomerulus is also globally sclerosed. (H&amp;E, ×400).

**A:**
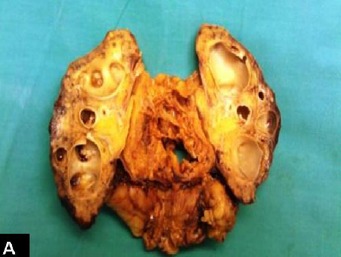

**B:**
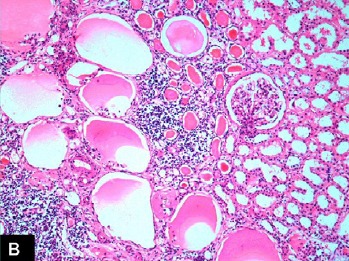

**C:**
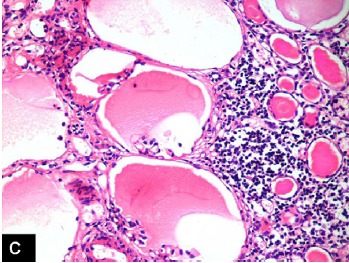

**D:**
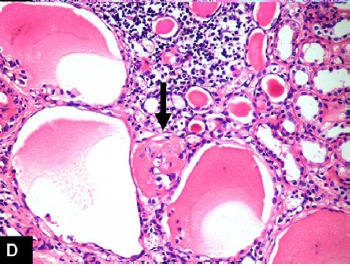

## Discussion

The term glomerulocystic kidney (GCK) denotes a kidney with more than 5% cystic glomeruli (1). The cystic glomeruli or glomerular cysts, in turn, are arbitrarily defined as Bowman’s space dilatation greater than 2 to 3 times normal size (2). It is widely acknowledged that the disease was first described by Roos (3), however, antecedent descriptions of the disease are also available (1). It is to be noted that GCK is not a single disease but rather it represents a phenotype that includes several diseases. The glomerular cysts are found in a variety of disorders, both hereditary and acquired, with equally varied clinical manifestations. In addition, there is a difference between GCK and GCK disease (GCKD), the two terms that are often used loosely and interchangeably. The later term should be reserved for the familial forms of GCK only (1). Indeed, the most important issue in the diagnosis of GCK is the exclusion of hereditary forms of the disease. However, ironically, the clinical, radiographic and histopathological features of both sporadic and familial forms are similar (1,4). Recent clinicopathological and molecular genetic studies have shed light on the etiopathogenesis of the disorder and its appropriate classification into clinically relevant categories (1).

GCK is more common in males (62%) and in children (72%) as compared with adults. However, the age range is wide from 20 weeks gestational age to 78 years (1-5). The condition can be asymmetric, unilateral or segmental in distribution (1). The present case most likely represents a unilateral disease associated with urinary obstruction and corresponds to type IV (obstructive GCK) as proposed by Lennerz *et al.* (1). This is supported by the normalcy of renal functions three years after the unilateral nephrectomy. The negative family history, absence of syndromic features, apparent unilaterality of the disease in an obstructed kidney and the adult onset all further corroborate this categorization.

## Conclusion

In conclusion, the diagnosis of GCK should not be considered the end result. It is imperative for both the pathologists and clinicians to categorize the lesion for its proper prognostication and appropriate management.

## Conflict of interests

None.

## Ethical considerations

Ethical issues (including plagiarism, data fabrication, double publication) have been completely observed by the authors.

## Author’s contributions

All authors contributed equally to the preparation of the manuscript.

## Funding/Support

None.
